# Early Detection of Anthracycline-Induced Cardiomyopathy: A Narrative Review of the Role of Artificial Intelligence and Remote Monitoring

**DOI:** 10.7759/cureus.111338

**Published:** 2026-06-23

**Authors:** Tosin T Fakiyesi, Oluwakemi Olalude, Olaoluwa Adeyemi, Olorungbami Anifalaje, Mmaduabuchi Ozioko, Oluwapelumi Ogunseye, Ibukunoluwa Adedoyin, Abiodun Adegbesan, Adeolu A Badejo

**Affiliations:** 1 Internal Medicine, Shirley Oaks Hospital, Croydon, GBR; 2 Internal Medicine, Lagos State University Teaching Hospital, Lagos, NGA; 3 Internal Medicine, Advocate Christ Medical Center, Illinois, USA; 4 Surgery, Mid Yorkshire Teaching NHS Trust, Wakefield, GBR; 5 Cardiology, Leeds Teaching Hospitals NHS Trust, Leeds, GBR; 6 Medicine, University of Ibadan, Ibadan, NGA; 7 Internal Medicine, University of Ilorin Teaching Hospital, Ilorin, NGA; 8 Institute of Medical Research, University of Tasmania, Tasmania, AUS; 9 Orthopaedic Surgery, Royal Victoria Infirmary, Newcastle upon Tyne, GBR

**Keywords:** anthracyclines, artificial intelligence, biomarkers, cardiotoxicity, echocardiography, heart failure, remote monitoring

## Abstract

Anthracyclines remain central to the treatment of many malignancies, yet their use is limited by the risk of anthracycline-induced cardiomyopathy (AIC), a complication associated with substantial long-term cardiovascular morbidity and mortality. The occurrence of cardiotoxicity during cancer treatment may adversely affect both treatment success rates and patient survival, especially when dose reduction or premature discontinuation of anthracycline therapy is necessitated, leading to treatment discontinuity, reduced therapeutic efficacy, and overall reduced quality of life. While anthracyclines improve cancer outcomes, the resulting cardiac damage may lead to heart failure, disability, repeated hospitalizations, and premature death, with the long-term cardiovascular complications often becoming a greater determinant of survival than the original cancer itself. Growing understanding of the biological mechanisms underlying anthracycline cardiotoxicity has improved the recognition of patients at increased risk and clarified the progressive nature of this condition, which typically evolves from subclinical myocardial injury to left ventricular dysfunction and, if unrecognized, overt heart failure. Risk stratification has traditionally relied on cardiac imaging and circulating biomarkers. Although some current surveillance methods for cardiotoxicity monitoring detect myocardial damage at subclinical and partially or fully reversible stages, many traditional methods are relatively late markers and detect myocardial damage usually after it reaches a stage where treatment becomes unable to reverse the damage. The limitations of left ventricular ejection fraction as a sole monitoring parameter have prompted increasing use of more sensitive imaging markers and biochemical indicators of early myocardial injury. This review conducts a medical evaluation of AIC to study triggers, patient manifestations, and risk factors that develop during treatment. It evaluates present diagnostic methods, including echocardiography, cardiac magnetic resonance imaging, and cardiac biomarkers, and investigates new methods that enable healthcare practitioners to detect diseases at their initial stages through artificial intelligence (AI)-based analytical tools, wearable technologies, and remote patient monitoring systems. Finally, we consider future directions aimed at earlier identification and personalized prevention of anthracycline-related cardiac dysfunction.

## Introduction and background

Anthracyclines are commonly used as chemotherapeutic agents in the management of solid tumors and hematological malignancies [1]. The intercalation of anthracyclines into DNA, inhibition of topoisomerase II, and generation of reactive oxygen species provide evidence of their antineoplastic effects [1]. Anthracycline-induced cardiomyopathy (AIC) is a potentially life-threatening side effect of anthracycline, characterized by a progressive myocardial injury, leading to left ventricular systolic dysfunction [2]. It involves topoisomerase IIβ-mediated DNA damage, oxidative stress, and mitochondrial dysfunction [3]. AIC can be traditionally classified by timing: acute (during or immediately after treatment), early-onset chronic (within the first year), and late-onset chronic (months to years), and this classification directs surveillance and management [3]. Currently, strategies for monitoring cardiotoxicity include imaging techniques (e.g., echocardiography), clinical biomarkers (e.g., troponins and natriuretic peptides), and emerging techniques (e.g., myocardial strain imaging) [4]. Although these methods are highly useful for detecting cardiotoxicity, their ability to detect early or subclinical toxicity is less established. Recent advances in AI offer the prospect of expediting early detection and risk stratification of AIC [5]. Applying machine learning (ML) and deep learning (DL) techniques can potentiate the identification of biomarkers of cardiotoxicity by analyzing multidimensional data, such as images, ECGs, clinical data from the electronic health record, and genomic information [6]. Additionally, time-dependent data and baseline data from patients may be analyzed to predict the development of cardiac dysfunction just before clinical symptoms emerge [7]. In parallel, wearable and digital technologies can monitor heart rate and rhythm and provide valuable data for patient monitoring [8,9]. However, evidence to support their routine clinical use is still emerging.

The aim of this present study was to examine the current evidence on AI and wearable technologies in AIC, explore their potential for early detection, review existing methods, and identify key areas where further research is needed.

Methods

A thorough literature search was conducted across principal electronic databases, including PubMed, Embase, and Scopus. Relevant articles between 2002 and 2025 were identified by using a set of predefined keywords, such as "anthracycline-induced cardiomyopathy", "cardiotoxicity", "artificial intelligence", "machine learning", "early detection", "biomarkers", "echocardiography", and "remote monitoring". Boolean operators, such as "AND" and "OR", were used. Studies that focus on detecting, risk-stratifying, or monitoring anthracycline-induced cardiotoxicity or AIC were included. The systematic search was restricted to original articles, systematic reviews, and meta-analyses published in English. The following study types were excluded from the analysis: articles that were primarily case reports, editorials/commentaries, conference abstracts without available full text, studies on anthracycline-induced cardiotoxicity treatment/management lacking relevant detection/monitoring information, non-human studies missing important mechanistic information relevant to the question, and duplicate studies. Articles that lacked sufficient detail regarding methodology were also excluded. Titles and abstracts were searched for relevance, and in addition, a full-text search was conducted for relevant articles. A manual search of the reference lists of the included articles was undertaken to identify additional studies. The findings were narratively synthesised with particular reference to emerging technologies and clinical applications. The study is a narrative review, and hence, no quality assessment or meta-analysis was performed. A total of 71 studies were included in the review of available evidence.

## Review

Anthracycline-induced cardiomyopathy

The pathophysiology of AIC involves interconnected molecular mechanisms, including oxidative stress, apoptotic pathways, and inhibition of topoisomerase IIβ, which together lead to progressive, cumulative myocardial damage [10,11]. Oxidative stress is an important contributor to cardiotoxicity [11]. Anthracyclines, via redox reaction, produce high reactive oxygen species (ROS) levels in the mitochondria of cardiomyocytes [12]. The myocardium becomes susceptible to oxidative damage because of its numerous mitochondria and weak antioxidant defenses [11]. Excess ROS causes damage to the mitochondrial DNA and cell membrane lipids, ultimately leading to a decline in cellular functions [11]. However, increasing evidence suggests that topoisomerase-IIβ (Top2β) inhibition is a pivotal and cohesive mechanism in anthracycline-induced cardiotoxicity [13]. The DNA damage response pathways become active because Top2β-anthracycline complexes form stable bonds with cardiomyocyte DNA, leading to double-strand breaks [13]. In addition, anthracyclines inhibit cardioprotective survival pathways, such as phosphoinositide 3-kinase/protein kinase B (PI3K/Akt) signaling, while simultaneously activating pro-apoptotic pathways, including p53 and mitogen-activated protein kinases (MAPKs) [14]. The interaction of these different mechanisms results in structural and functional cardiac changes [11]. Considering this mechanistic complexity, risk factors for AIC arise from the interaction between patient-specific vulnerabilities and treatment-related exposures, leading to variability in cardiotoxic risk among patients undergoing anthracycline chemotherapy [15]. Identifying these risk factors is necessary for effective risk stratification, surveillance planning, and cardioprotective intervention [15]. Cumulative anthracycline dose is an important risk factor to consider, as even cardiotoxicity may manifest at considerably lower doses, especially in susceptible patients [15]. AIC is dose-dependent, with the cumulative dose threshold of doxorubicin-equivalent dose ≥ 250 mg/m², a cutoff used by the National Comprehensive Cancer Network (NCCN), American Society of Clinical Oncology (ASCO), and Heart Failure Society of America (HFSA) [16]. The risk of heart failure increases in a dose-dependent fashion with doxorubicin, from 5% at 400 mg/m² to approximately 48% at 700 mg/m², signifying an almost tenfold increase in risk [17]. Despite the recommended threshold, morphological changes have been observed at doses as low as 200 mg/m², with troponin elevations observed after a single treatment [18]. Age is another important consideration; older adults may be at increased risk due to age-associated reductions in cardiac reserve, mitochondrial efficiency, and endogenous antioxidant capacity [19]. The pediatric population is more susceptible to cardiotoxicity even at lower cumulative doses with a prolonged risk window extending several years after treatment [20]. In a systematic review of 30 studies involving 12,507 children, a maximum dose > 45 mg/m² given within one week was associated with higher rates of anthracycline-induced clinical heart failure (A-CHF) [21]. Another concern in the pediatric population is anthracycline-induced impairment in myocardial growth in the developing heart, resulting in reduced left ventricular wall thickness and increased wall stress, which makes the heart susceptible to secondary stressors, such as hypertension, that may precipitate heart failure in early adulthood [20]. Cardiovascular comorbidities, such as hypertension, diabetes mellitus, dyslipidemia, and obesity, can worsen susceptibility to AIC by promoting endothelial dysfunction and chronic inflammation [15]. Clinically, AIC often follows a variable and sometimes insidious course. Many patients remain asymptomatic in early stages, with subclinical myocardial injury before the emergence of left ventricular dysfunction marked by symptoms such as fatigue, dyspnea, orthopnea, and peripheral edema [22]. However, progression is heterogeneous, and timely detection remains crucial, as early intervention can significantly improve outcomes. Initiating cardioprotective pharmacotherapies, such as beta blockers and angiotensin-converting enzyme inhibitors, at the onset of asymptomatic cardiac dysfunction can enhance systolic function, reduce ventricular remodelling, and decelerate the progression to heart failure [23].

Limitations of conventional detection approaches

According to guidelines from the American College of Cardiology, surveillance for patients undergoing anthracycline-based treatment who are termed to be high risk includes imaging and biomarker evaluations every three to six months during treatment and annually for ≥ 5 years after treatment [24]. The European Society of Cardiology suggests that cardiac biomarkers should be used throughout a cancer patient's treatment pathway, with intensity of use adjusted based on the patient's estimated baseline cardiovascular risk [15]. Additionally, echocardiography is the imaging modality of choice for the evaluation of such patients with baseline left ventricular ejection fraction (LVEF) as an index of systolic function and predictor of future heart failure [10]. A comprehensive baseline assessment is to be done to determine the baseline risk of each patient prior to commencement of therapy [15].

*Biomarkers* 

Biomarkers are measurable blood-based indicators of underlying cardiovascular processes and are useful in assessing heart-related toxicity during cancer therapy [25,26]. In clinical practice, cardiac troponins (cTn) and natriuretic peptides (NPs) are the most commonly employed. cTn are sensitive markers of ongoing myocardial injury and have proven effective in identifying patients with a heightened risk of subsequent left ventricular failure following anthracycline treatment [27,28]. NPs, including B-type natriuretic peptide (BNP), signify myocardial wall stress and may provide additional insights into the advancement of cardiac dysfunction [29]. These indicators detect myocardial injury, which may have been present and ongoing prior to the commencement of anthracycline-based therapy; hence, their ability to accurately predict therapy-based cardiotoxicity may remain limited. Some studies indicate variability in BNP levels among individuals, which may influence interpretation within particular ethnic groups [30], with a study by Gupta et al. revealing lower concentrations of NT-proBNP in Black and Chinese individuals, thereby supporting this concept [31].

*Imaging* 

Cardiovascular imaging is important at baseline and during follow-up for patients on anthracyclines [25]. Cancer therapy-related cardiac dysfunction is defined as a decrease in LVEF of ≥ 10% from baseline to a value below 50% [32]. However, using LVEF as an endpoint has several limitations. Changes in LVEF generally develop late in the myocardial injury process and reflect a reduction in myocardial contractility after substantial degrees of myocardial damage have occurred. Therefore, endpoints that focus on more acute indicators of injury, such as viable myocardium identified by contractile reserve or by inward motion during LV contraction, as evidenced by the left ventricular circumferential motion (LVCM), may offer more sensitive assessments of myocardial salvage [29]. Myocardial strain imaging, in particular, global longitudinal strain, is a more sensitive marker of subclinical myocardial damage than LVEF. Decreased strain values can even precede decreased LVEF values for months, thereby allowing the detection of cardiotoxicity at an earlier stage [33]. Cardiac magnetic resonance imaging (CMR) continues to be the most reliable method for evaluating cardiac morphology and function [34]. However, availability, cost, longer acquisition times, and patient-related factors such as claustrophobia and contraindications to contrast agents limit its use [35].

Need for Continuous, Predictive, and Personalized Monitoring Approaches

Due to the significant variability in clinical presentation and severity of CV toxicity, a personalized approach to CV toxicity management that takes into account a patient’s individual factors, such as genetic risk and exposure to toxic agents, is necessary ​[36]. Biomarkers, most notably cTn, have recently been described as useful tools for risk stratification and monitoring of cardiac function in patients with suspected or confirmed CV toxicity. However, more sensitive monitoring methods are still required [37,38].

Artificial intelligence (AI) in early detection 

Overview of AI, Machine Learning (ML), and Deep Learning (DL) in Cardio-Oncology

AI, ML, and DL have been explored for cardiovascular risk stratification and early disease detection across several conditions [38]. These tools can also be used in cardio-oncology to identify patients at risk of AIC before current screening protocols detect late-stage cardiotoxicity. The integration of an AI-based decision-support system into the diagnostic workflow enables data-driven analysis of a large number of parameters using structured data inputs [39]. Such an approach can be particularly advantageous in cardiology, where AI can rapidly evaluate ECGs, imaging studies, and patient records to identify early signs of myocardial damage [6]. In practice, AI has shown potential to detect subclinical left ventricular dysfunction and stratify cardiovascular risk. Most applications are still in an experimental validation phase [40].

Although AI has shown promise in many fields of medicine, including oncology [41], a thoughtful approach should be taken when applying it to the challenges of detecting and predicting anthracycline-induced cardiotoxicity. The majority of AIC studies to date are retrospective or small, relatively homogeneous patient studies and, therefore, may not serve as adequate surrogates for other oncology populations [38]. While other fields and medical applications can provide general lessons for AIC methodology [42], they should not be over-interpreted for this specific problem. AI could facilitate early detection of cardiotoxicity, enhance risk stratification, and guide clinical decision-making. Prospective validation of current approaches and the development of disease-specific models are, however, required before they can be safely introduced into routine cardio-oncology practice. Advances in AI have enabled the integration of AI technologies in surveillance to detect AIC early. A recent study used a previously validated AI-based ECG analysis approach. Researchers examined serial 12-lead ECGs collected prior to and subsequent to the commencement of anthracycline therapy. They calculated a probability score for reduced LVEF (≤ 40%) [43]. The study indicated that conventional echocardiography could detect cardiac dysfunction almost a month earlier than it actually does. However, these results should be interpreted with caution because the study population was small and a specific group was selected [43]. Verifying the performance of previous cardiotoxicity markers in a larger patient cohort would be desirable.

Detection of Subclinical Cardiac Dysfunction

Recent findings suggest that AI could help detect very early signs of vascular changes. Some AI models for analyzing cardiovascular parameters can detect early signs of increased cardiovascular disease risk in conditions (e.g., diabetes, obesity, and rheumatoid arthritis) associated with systemic inflammation and endothelial dysfunction [44]. In AIC, the same principles may be applied where early myocardial damage is caused by oxidative stress and changes in the microvascular circulation. Recent studies have found that AI can improve screening accuracy for subclinical cardiovascular disease in asymptomatic individuals across multiple data dimensions, including ECG, imaging, and clinical data [45]. In addition, AI-enhanced imaging has high accuracy for early detection of coronary and myocardial changes, but must be considered on an individual basis and in specific clinical circumstances [46]. However, the use of such an approach in AIC remains at an early stage. Most available information has been derived from studies performed on populations apart from patients with severe oncologic illnesses or studies conducted in research settings. Consequently, AI may serve as a screening tool to detect early signs of potential cardiotoxicity; however, the results must be interpreted with caution and correlated with established indices of cardiotoxicity [45]. Recent AI models for various healthcare tasks are moving away from simply achieving optimal performance and instead aim to provide clinically interpretable risk predictions. In healthcare, it is crucial to explain how variables affect the final score or decision, thereby increasing trust and the usability of AI models [47]. Similarly, AI models trained on large volumes of electronic health record data have demonstrated the ability to predict clinical deterioration and provide clinicians with a more profound understanding of the models' underlying decisions [48]. Different models have been proposed to predict early cardiotoxicity in AIC using a combination of ECG, imaging, and biomarkers. Most models require further validation and should be used as adjunct measures with cautious interpretation [48].

Remote monitoring and wearable technologies

Real-time cardiovascular system monitoring using wearable devices is emerging. These devices, such as smartwatches, fitness bands, and smart rings, monitor heart rate, cardiac rhythm, physical activity, and, in some cases, blood oxygen levels using photoplethysmography (PPG) [49]. In addition, some wearables even incorporate cuffless blood pressure estimation using optical or tonometry-based signals. However, such estimates are non-trivial to implement and require repeated calibration [50]. Furthermore, the estimation of hemodynamics using wearable sensors is in its infancy; findings are conflicting, and the evidence supports a cautious interpretation of measurements [51]. Telemonitoring systems integrate data from wearable devices, mobile health applications, and self-reported information to facilitate long-term patient surveillance and enable early clinical review following a deviation from baseline [52]. AI analyzes large datasets of time-related information collected by these devices to identify evolving patterns and subtle deviations that current monitoring approaches may not detect [53]. Evidence shows that improved monitoring and management of cardiovascular risk can prevent adverse outcomes in the general population. While this assertion is less certainly true for the application of better monitoring and management of cardiovascular risk to the wearable-based detection of anthracycline-induced cardiotoxicity, it remains an area of interest that requires validation [54]. In parallel, advances in DL continue to expand AI's potential in cardiovascular medicine, especially for identifying complex patterns in large datasets using multimodal inputs [55]. Remote monitoring enhances patient convenience and facilitates continuous monitoring of cardiac device data; however, its current application in cardio-oncology is restricted to complementing, rather than supplanting, established monitoring strategies. Remote monitoring can be integrated into clinical workflows thoughtfully, considering existing clinical infrastructure, data governance, and implementation strategies to optimize its use in clinical practice [56].

Integration of AI and remote monitoring

Recent studies have explored the integration of AI into remote patient monitoring systems to enhance cardiovascular risk assessment in patients receiving cancer therapy. Utilizing data from multiple modalities and sources of varying types, such as electrocardiograms, physical activity, laboratory values, and patient-reported outcomes, a new class of AI monitoring systems, such as CardioAI, can perform real-time monitoring of cancer therapy-induced cardiotoxicity [57]. However, these systems are primarily investigational at this time and require further clinical validation. AI in remote monitoring primarily interprets data rather than collects it, mining useful patterns and models from continuously collected inputs [58]. The value of AI in monitoring applications lies in its ability to contextualize and analyze complex, longitudinal data over time from individual patients or large datasets. Despite the immense potential of AI in healthcare, ongoing monitoring and generated alerts present significant operational challenges. A structured triage approach could help clinicians identify and focus on clinically important signals while hiding less important ones to avoid alert fatigue. This would let clinicians do their jobs well within their current workflows and roles [59]. Moreover, human-AI interaction models have been proposed to support optimal decision-making. In such approaches, AI can be employed as a tool to support clinical decision-making. AI results are most effective when used to supplement and enhance clinical diagnosis rather than serve as a substitute for it [60]. Implementing AI-based clinical decision support systems (AI-CDSS) in real-world clinical settings is critical for their effective use. Human-centered design, or ergonomics, has recently been identified as a set of important principles for improving the usability and interpretability of systems developed for clinical use and ensuring that the system's design fits the clinical workflow in which it will be used [61]. From a systems perspective, remote patient monitoring platforms can utilize AI models as part of a system that processes multimodal data collected from different sources [62]. Personalized monitoring algorithms can track vital signs and monitor deviations relative to an individual's baseline, rather than against general population statistics [63]. Despite progress, there are still some problems that make these systems less useful in the clinic and harder to scale. Chief among these limitations is the dependency of current systems on high-quality, reliable, and complete data, which is highly sensitive to factors such as device usage and patient engagement. Additional challenges include the impact of usability, data integration, and clinical workflow integration on their scalability and clinical usability [64].

Research gaps and future directions

Substantial variability exists across data sources, model development, and reporting standards [65-67]. Future models must incorporate standardized frameworks for development and validation, as well as for clear, transparent reporting, to aid clinical application and achieve regulatory approval. While there are important advances in the development and clinical utility of AI in cardiovascular medicine, there is a further unmet need with respect to the evaluation of current AI models in terms of external validity and equity, particularly with respect to the representation of various populations, such as children and older adults, and those from different ethnic groups [68]. The evaluation of equity in future models is particularly important in the emerging field of cardio-oncology, where patient risk profiles differ substantially from those of the general cardiovascular patient population.

A major challenge for existing methods is their validation in large, multi-center, prospective studies across different patient groups. So far, most studies have been conducted as retrospective analyses or in single-center studies, both of which are prone to bias [43,69]. Large-scale prospective validation studies are needed to determine whether AI-assisted strategies can help achieve clinical goals, such as early detection or prevention of progression to heart failure, in patients with anthracycline-induced cardiotoxicity, particularly given its variable course. A lack of integration is observed among ethical, regulatory, and clinical practices related to AI in healthcare [70,71]. While progress is observed in data governance, model explainability, and evaluation within controlled environments, there is a need for further work to address monitoring, oversight, and safe clinical deployment in real-world practice. Future work should focus on developing and validating frameworks to address these clinical needs.

## Conclusions

AIC is a severe cardiac side effect of cancer chemotherapy that is dose-dependent and agent-specific. Its mechanism is complex and involves oxidative stress, mitochondrial dysfunction, and topoisomerase IIβ-induced DNA breaks leading to myocardial injury. The clinical course of AIC is unpredictable and highly variable. Early diagnosis may reveal a degree of reversible myocardial injury. Current clinical strategies for monitoring myocardial stress rely on echocardiography and the measurement of cardiac biomarkers, such as troponin. These methods have limitations, particularly in sensitivity for detecting early or subclinical alterations in myocardial function induced by stress. Consequently, clinical monitoring frequently identifies significant myocardial injury and substantial declines in left ventricular function but may overlook more subtle effects. Improved monitoring strategies for myocardial stress remain an evolving field of research. Early diagnosis and patient-specific monitoring of adults with AIC may be achieved by implementing AI that integrates data from multiple clinical domains, such as ECGs, images, biomarkers, and electronic health records. While there is some evidence for the use of AI in clinical settings, more research is necessary. Future research on AIC will focus on developing and validating novel AI-based detection strategies for stressed myocardium, as well as increasing the interpretability of current and future AIC models to integrate AIC into the current healthcare system.
